# Detecting Proximity with Bluetooth Low Energy Beacons for Cultural Heritage

**DOI:** 10.3390/s21217089

**Published:** 2021-10-26

**Authors:** Paolo Barsocchi, Michele Girolami, Davide La Rosa

**Affiliations:** ISTI-CNR, Institute of Information Science and Technologies, 56124 Pisa, Italy; michele.girolami@isti.cnr.it (M.G.); davide.larosa@isti.cnr.it (D.L.R.)

**Keywords:** location-based services, BLE beacons, proximity detection, ambient intelligence, cultural heritage

## Abstract

The RE.S.I.STO project targets visitors of Pisa medieval city, with the goal of providing high-quality digital contents accessible with smart devices. We describe the design, implementation and the test phases of the RE.S.I.STO application, whose goal is to automatically detect the proximity between visitors and artworks. Proximity is detected with a set of algorithms based on the analysis of Bluetooth Low Energy beacons. We detail our experimental campaigns which reproduce several museum layouts of increasing complexity at two pilot sites, and we compute the performance of the implemented algorithms to detect the nearby artworks. In particular, we test our solution in a wide open space located in our research institute and by performing a real deployment at the Camposanto Monumentale located in Pisa (Italy). The obtained performance varies in the range of 40% to perfect accuracy, according to the complexity of the considered museum layouts. We also describe a set of stress and stability tests aimed at verifying the robustness of the application during the data collection process. Our results show that the mobile application is able to reduce the beacon loss rate, with an average value of 77% of collected beacons.

## 1. Introduction

With the growing number of IoT-ready devices and their rapid diffusion in our daily lives, in the last decades, we witnessed the pervasive adoption of mobile apps delivering location-based services [1]. The possibility of determining the location and the proximity of a user represents a key-factor to deliver context-aware services, thus improving the user-experience [2].

Therefore, in the last few years, a growing interest has been observed in research topics addressing the estimation of the location of mobile users both indoor and outdoor. This goal still represents a challenging task for indoor scenarios, where solutions based on Global Navigation Satellite System (GNSS) are unfeasible. Estimating the location of a target requires the adoption of technologies and data analytic tools that most often cannot exploit satellites as walls, wireless signal interference and obstacles significantly reduce the strength of satellite signals in indoor settings. To overcome this issue, in the last years several methods have been proposed, exploiting different kinds of wireless signals and technologies. Furthermore, the design of indoor proximity/localization solutions strictly depends on the target service. For example, the accuracy required to identify the occupancy of a room might be far lower than that required to guide a robot along a path. As a matter of fact, there is no *standard de facto* localization solution for indoor spaces (similarly to GPS), leading to a still active and attractive research question.

Indoor proximity detection systems are of remarkable importance since they allow to provide the users with space-bounded services in a wide number of scenarios, such as health sector, industrial safety, urban mobility, emergency management, contact tracing, etc. For what concerns cultural environments such as museums or exhibitions, proximity detection systems can provide the users with context-aware information based on their closeness to points of interest thus improving both their experience and entertainment. To be easily adoptable in this kind of environments, proximity systems should be based on widely diffused technologies, be easy to use for the visitors, require a straightforward setup phase, provide high level of effectiveness and the user devices should be able to work for sustained periods of time on battery power. Despite that a multitude of techniques are available in the literature, they always entail a trade-off among those aspects. These challenging tasks keep this research field open to new approaches which can provide further analysis in real experimental scenarios.

In this paper, we design and test a mobile cross-platform application, based on the Bluetooth Low Energy (BLE) technology, to automatically detect the proximity between visitors and points of interest, e.g., museum’s artworks. In turn, such application will be adopted to provide to visitors the correct digital content of the available artworks. Some remarkable works already addressed the problem of localizing people in a museum or to estimate their proximity with artworks, we refer to [3,4,5] for further details.

Our goal is to be able to correctly estimate the proximity with artworks without any prior knowledge about the followed path. To this purpose, we describe in this paper the design and test of the RE.S.I.STO app. We implement two different proximity detection algorithms and we compare their performance in terms of a classification problem in two pilot sites. More specifically, we measure how much our algorithms are able to correctly identify the nearby artworks against a detailed ground truth. Each artwork is equipped with a Bluetooth tag that emits BLE signals at periodic intervals with a pre-defined power of emission. Our tests also include a stress and a stability assessment of the performance of the app. In particular, we quantitatively measure the beacon loss rate at different conditions with the goal of measuring the amount of collected beacons with respect to the expected number of beacons. More specifically, we set up stress and the stability tests: stress tests measure the maximum number of collected beacons for a relatively short time period. Differently, stability tests aim at measuring the amount of collected beacons for a long period. We compute the beacon loss rate, as the ratio between collected and expected beacons averaged with respect to the different runs of each test typology. Our experimental results return an average of 77% of expected beacons with commercial smart devices and we reach an accuracy and F1 score varying between 100% and 40% according to the type of museum layout and of the pilot site. We report below the main contribution of this work:

- The performance assessment of two proximity detection algorithms based on the RSS analysis of the collected beacons. More specifically, we compare the results in two pilot sites with different museum’s layouts of increasing complexity and we show how the performance varies when the target artwork is identified within the first, second and third option;
- The analysis of RSS’s fluctuations in which we study two key-metrics: the beacon loss rate measured with commercial BLE tags and smartphones and the RSS variation in indoor environments caused by the usage of three advertisement channels. Under this respect, it is worth noticing that we propose a possible strategy implemented in the RE.S.I.STO app to limit the side-effect of such frequency hopping based on the use of a specific quantile of the beacon’s RSS values.

The paper is organized as follows: Section 2 reports the Related Work organized according to different proximity technologies. Section 3 describes the design of both the mobile app and the proximity algorithms. Section 4 details the experimental settings, the testing session and the performance assessment of the implemented algorithms.

## 2. Related Work

Digital guide applications are available for most museums worldwide. However, solutions designed to deliver recommended contents based on the user’s proximity with artworks are much less frequent [6]. By exploiting the smartphones capabilities existing today, several approaches are available to implement artwork proximity detection systems which could be broadly classified into three main groups: radio-frequency-based, vision-based and others (e.g., infrared, ultrasonic). Each of these techniques have benefits and limitations that are discussed hereafter.

We report in Table 1 a summary of the technologies we considered for the purpose of this work. We report their limitations and the advantages and the list of those works adopting such technologies.

### 2.1. Radiofrequency-Based Techniques

Bluetooth Low Energy (BLE) beaconing is a technique based on compact wireless devices (tags) able to periodically broadcast, in the surrounding environment, a radio signal containing a predefined message. This procedure is called *advertisement* and the transmitted packets can be received from any BLE-enabled device, which are today extremely diffused. Several works are based on BLE beaconing such as [7,8,9]. In [10], BLE beacons are used to implement a system for proximity target marketing based on the iBeacon advertising messages. These approaches, based on the Received Signal Strength (RSS), allow a rather precise proximity estimation, especially, when beacons are individually calibrated. The main limitations include the need to physically deploy the BLE tags in the environment and the fact that the RSS could be affected by both the museum crowd and the signal reflections caused by wall or obstacles. Tags count, position, transmission frequency and power have to be tuned, during the preliminary study phase, to reduce the side effects.

WiFi is a common solution for avoiding heavy infrastructures, as the existing WiFi access points (AP) are exploited for the localization and proximity purposes. Such solutions do not require that the user device and the AP are in line-of-sight, rather a combination of lateration and fingerprinting techniques can be used to obtain enough accuracy [6]. In [11], the authors present a technique for evaluating proximity detection through WiFi in mobile web-based applications that requires zero configuration on the client device. Although the proposed approach is low-cost and easy to implement, it lacks in accuracy since it cannot directly estimate the distance of the user from the artwork. SmARTweet [12] is a smart multimedia guide and a location-based application developed to automatically suggest contents to the visitors by letting the artworks “tweeting” their story to the users. The system, although quite promising, requires the deployment of many WiFi anchor nodes in the environment requiring main power and maintenance. Further WiFi approaches, based on RSS fingerprinting, are presented in [13,14]. Fingerprinting requires a prior offline phase during which a database, containing the RSS of every detected AP in several locations, is built. For very large environments such as museums, this technique could be burdensome due to the extensive preliminary data collection campaign that has to be carried out. We also mention the use of WiFi probes to detect the proximity as done in [15]. These authors deploy a number of WiFi probe receivers and analyze the WiFi probes emitted by commercial smartphones for the localization purpose.

Ultra-wideband (UWB) is a radio technology for transmitting very short pulsed messages that are spread over a large radio spectrum. UWB-based systems employ the trilateration approach which can estimate the position of a device within few centimeters of accuracy and do not require a line-of-sight path between the transmitter and the receiver, even though, in this latter case, the performance deteriorates. The works presented in [16,17] explore this approach in museum settings. UWB is the most accurate ranging system available today for indoor scenarios [18] but requires an extensive infrastructure setup with several UWB anchors deployed in the environment and, to date, compatible end-user devices are still not very diffused on the market.

Near Field Communication (NFC), based on RFID close-range contactless connection, is another technique used for proximity detection. These systems use the radio waves emitted by a reader (smartphone) to detect and query the data stored on RFID tags which are embedded in compact enclosures such as buttons or cards. Several works such as SMART VILLA [19], Wolfsoniana Smart Museum [20] and iMuseumA [21] proposed a proximity detection approach for museum scenarios. NFC, although very diffused on today’s smartphones, has a sensing range typically limited to a couple of centimeters, which restricts the possibilities of interaction, especially in museums where spaces might get very crowded.

We further analyze the BLE-based works we previously surveyed with the purpose of highlighting the distinguishing features of such systems running in a museum environment. To this purpose, we report in Table 2 a comparison of the selected BLE proximity solutions evaluated against seven criteria describing the complexity behind realistic experimental tests in indoor environments:

- Realistic scenario: we analyze if the solutions have been tested in a real museum or in a simplistic environment;
- Device heterogeneity: we report if authors tested their solution with a variety of devices or, differently, if only a specific device model has been used, e.g., ad hoc hardware. This aspect is crucial for the performance evaluation as BLE-based solutions might be affected by different BLE chipsets estimating the signal strength of BLE messages differently;
- Complex path: the complexity of the path followed to test the proximity with points of interests also affect the overall performance. We analyze if authors selected a trivial or nontrivial experimental path;
- Commercial devices: we further analyze the device adopted in order to report if commercial devices have been adopted;
- Robustness tests: we report if the proposed work also provides information about the robustness of the proposed solution. More specifically, we are interested in solutions working with long-lasting monitoring sessions, reproducing a realistic museum visit;
- Preliminary RSS data analytics: we analyze if the works provide a preliminary RSS analysis of the collected data so that to characterize the signal’s features of the BLE messages used to estimate the proximity. This analysis is important to better understand the indoor signal propagation for the considered testing scenario;
- Real-time outcome: we finally analyze if the solutions are designed for a real-time proximity estimation or for off-line evaluation. In the first case, the solution can notify to the user the proximity with a specific point of interest, while, in the second case, the proximity is detected off-line.

In [7], the authors propose a system to gather information about the behavior of museum visitors. The solution was deployed at the Cobra Museum of Modern Art (NL) and tested with a realistic path involving several rooms and artworks. The devices used are only ad hoc mobile nodes worn by the visitors and ad hoc anchor points placed in the rooms. No robustness tests have been performed since the device performance is already known. The system processes the data offline to provide a service to the museum manager and exploits a filtering pipeline to handle the bursty and noisy raw data collected. The system proposed in [8] was also deployed in a real world scenario such as the Louvre Museum (France) and exploits the beacons collected from the visitors personal devices to track the visiting path. The monitored route is realistic since it comprises several spaces within the museum even at different floors. The authors are using customized Bluetooth sensing devices and no robustness tests nor preliminary RSS analysis are reported. The system is designed to provide offline results to highlight the visitors most covered routes. In [9], the authors present a BLE Direction-of-Departure-based system able to exploit only the RSSI observed at the receiving terminal. The experimental results are obtained in a lab setup consisting of a single room. A laptop is used as the receiving device and few positions at the center of the room are tested for a very short period of time. The transmitting BLE device is a custom-made node with a prototype antenna to generate different radiation patterns. A preliminary RSS analysis is reported in order to perform the real-time triangulation technique using DODs from multiple beacons. In [10], the authors explore the iBeacon message format to propose a proximity detection application that, although designed for marketing purposes, can be used in the museum scenario. The proposed solution was demonstrated in a lab environment by using a custom-developed BLE beacon transmitter and an iOS device running the proximity detection application. No robustness tests nor preliminary RSS analysis are reported. The application is able to estimate the proximity to a point of interest in real time and provide the user with the detailed product information. The solution proposed in [18] exploits the Estimote commercial tags to analyze the received RSS signal and uses the path-loss model formula to apply the ranging approach. The system is tested in a corridor in laboratory conditions, a single device model has been used and few points of interest, placed on the walls, are tested. Although the system robustness test is not reported, a preliminary RSS analysis is performed to fit the path-loss model to the environment and correct the bias due to noisy RSS measurements. The algorithm is able to estimate the output in real-time. Eventually, in [30], the authors propose a mobile application, exploiting the commercial Gimbal Series 21 beacon to estimate the proximity to points of interest by implementing a ranging algorithm supported with a Kalman filter. The results are obtained in a laboratory environment consisting of a room and a corridor. A single smartphone model is used as the receiving device and, even in this case, few locations are tested. The developed application is able to generate the proximity outcome in real-time although no long duration robustness test is reported.

With our proposed solution we try to address all these critical aspects and provide a robust and commercially available architecture ready to be deployed in a real world scenario.

### 2.2. Vision-Based Techniques

Vision-based techniques rely on the detection of both peculiar image features, such as points, edges or regions, or on visual two-dimensional pattern such as bar codes or QR codes [24]. To acquire the images, cameras can be mounted on smartphones or wearable models provided to the visitors. Artwork image detection has been proposed in [22,23], these approaches require a training phase and are susceptible to partial visual obstruction caused for instance by a crowded environment. In [23], the authors present an approach for image retrieval and object detection based on fully convolutional networks but it is particularly targeted at small datasets with low object variability. Moreover, it should be taken into account that museums are complex environments, which might expose plenty of artworks, and each of them requires to be classified from different perspectives, distances and lighting conditions [6].

QR or bar codes represent a cheap technology and the physical tag is able to store enough data, such as an ID or a website URL, to fetch the description of the artwork from the back-end server. However, in a museum environment, the usage of QR codes might not be a viable solution since they both interfere with the artistic visual and they require the user to take a photo, which is often prohibited in this kind of scenarios [25].

### 2.3. Other Techniques

The authors of [26] present an ultrasonic indoor location tracking system for mobile devices based on off-the-shelf audio speakers, while in [25], the authors developed a smartphone museum guide that identifies surrounding artworks by receiving a modulated ultrasonic signal. Their approach requires no special hardware on the receiver side, and the sound signal can be generated via cheap stand-alone devices. The main limitation of these approaches is that they require an infrastructure to be deployed to generate the ultrasound signals. Another technique, mainly used in early systems, is based on the infrared signals and employs beacons emitting a modulated IR light based on a unique identifier code [27,28,29]. The emitted beams have several limitations since they are highly directional, require line-of-sight and many devices are not equipped with IR transceivers.

As reported in Table 1, many solutions for proximity detection have been proposed. We observe that in many cases the conducted experiments are limited to prototypes tested with dedicated software and hardware components. In this work, we try to further explore this research domain by focusing on two main aspects. On the one hand, the adoption of a cross-platform framework able to abstract from the underlying mobile Operating System. On the other hand, we analyze the performance and the collected data at two pilot sites reproducing realistic conditions both in a laboratory and in a real scenario. We assess the platform beacon loss rate and we address the frequency hopping behavior of the BLE advertising by adopting a methodology to mitigate such fluctuations based on the use of a specific statistic of the collected RSS values.

## 3. Application Design, Tools and Methods

We now describe the design of the RE.S.I.STO app with specific attention to the technology used and the proximity detection algorithms that we implemented and tested.

### 3.1. The Design of the RE.S.I.STO App

The RE.S.I.STO app implements a multi-platform mobile application based on the React Native Framework (see Section 3.2). The application is designed to automatically detect the proximity between visitors and a number of points of interests available in a region, for example, artworks in a indoor museum. The app is based on three services, namely:

- the Beacon Logger Service (BL);
- the Proximity Detection Service (PD);
- the UI Content Viewer Service (UI).

**Figure 1:**
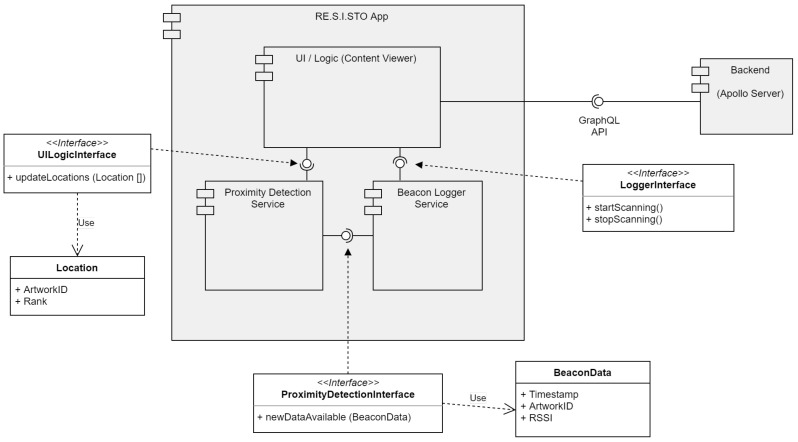
Design of the RE.S.I.STO app.

The cooperation between the three services is implemented with a set of API. We report in Figure 1 an overview of the design of the RE.S.I.STO app. The BL module is designed to continuously gather Bluetooth beacons for the proximity detection purpose. BL is designed to collect the maximum number of beacons emitted by nearby tags, without negatively affecting the performance of PD and of the UI services. Moreover, the BL service filters beacons emitted only by the tags belonging to our experiment, avoiding gathering data from unrelated devices. Filtering the collected beacons reduces the overhead of the application and, at the same time, it allows to increase the performance of the PD and of the UI service. The BL service reacts as soon as new beacons are collected, and it performs the following operations:

- Parsing the beacon payload;
- Extracting the information required for the PD service;
- Notifying the PD service with a set of the received beacons.

**Figure 2:**
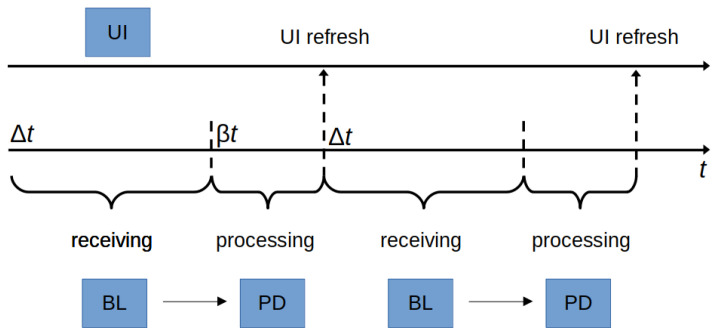
Interactions between the BL and PD services.

BL interacts with the PD service by invoking the ProximityDetectionInterface. PD elaborates the received information, by running a suite of proximity detection algorithms (see Section 3.3 for a description of the implemented algorithms). More specifically, the beacon’s information is elaborated at periodic intervals. We report in Figure 2 how the BL and the PD services interact. BL listens for beacons in a time window of Δt seconds, after which it invokes PD for processing the collected information. The interaction between PD and UI is implemented through the UILogicInterface.

**Figure 3:**
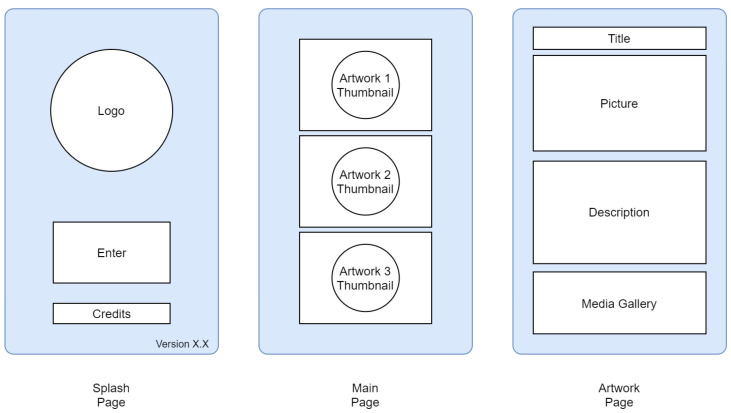
Blueprints of the three views composing the app UI flow.

The UI service visualizes the information elaborated by PD, by showing the closest *k* tags in proximity. The UI service refreshes the information as soon as PD finishes its computation, as shown in Figure 2. The UI flowchart is reported in Figure 3, it comprises three screens: a splash page, the main page with the set of the top *k* tags and the artwork page with a detailed description of the information associated with the selected tag.

Finally, our design comprises an external database to fetch the information to be displayed by the UI service. We refer to it as the back-end module, as shown in Figure 1. The back-end can be queried with GraphQL API to download the meta-information associated with the selected tag.

### 3.2. The React Native Framework

**Figure 4:**
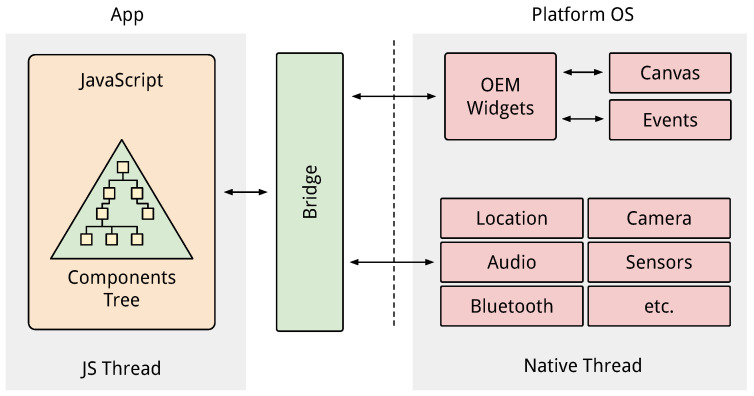
Overview of the React Native architecture.

React Native is an open source Javascript framework used to develop native mobile applications running both on iOS and Android operating systems. The framework is based on the Facebook’s widely used Javascript library, that targets web browsers, called React. The main advantage of React Native is the portability of the code among the current mobile platforms, allowing the creation on mobile apps that look, feel and perform like real native apps rather than typical web apps. The basic concept underneath the portability is the Virtual Document Object Model (Virtual DOM). The DOM is the logical tree structure that describes all the elements in a page while the Virtual DOM is a layer of abstraction on top of the DOM which allows to translate abstract components to platform-specific native components during the rendering phase. The translation is performed by the so-called render bridge, which talks to the platform’s native API to generate the native components and render them on the screen. The bridge sits between two other main components: native modules and the Javascript virtual machine. These two components run in separate threads and they communicate through the bridge as shown in Figure 4. During the app execution, the code running within the JavaScript thread invokes the bridge component to perform instructions (e.g., creating components, showing views, etc.) on the native modules. The native modules, in turn, satisfy the request and subsequently acknowledge the completion of the task back through the bridge to the Javascript thread [31]. React Native allows to reduce the development time by:

- Providing a large and ready to use component repository;
- Reusing at most the codebase to target both iOS and Android platforms;
- Allowing the hot reload of the app when modified without the need of compiling every time.

Concerning the performances, although Javascript is not as fast as native code, differences will not be noticeable in most cases. Moreover, the most demanding code could still be moved to a native module to remove the performance bottlenecks. On the negative side, React Native requires a not negligible effort to setup the initial development environment and might expose compatibility, dependency and debugging issues, especially, when dealing with some platform-specific modules. Overall, to develop cross-platform UI rich apps, it is among the most indicated platforms.

### 3.3. Proximity Detection Algorithms

In this subsection, we describe the algorithms we implemented to detect proximity between visitors and artworks. Such algorithms have been integrated with the RE.S.I.STO app. The objective of these algorithms is detecting the proximity between visitors and artworks in historical museums. More specifically, we do not track the position of the visitor in real-time, as generally done with indoor localization algorithms. This helps us to avoid one of the classical issues in this kind of algorithms such as a time-consuming calibration phase.

Most of the commercial devices natively allow estimating the strength of Bluetooth signals emitted by tags, this measure is generally referred to as Received Signal Strength (RSS) and it is expressed in dBm units. Our algorithms rely on the RSS values collected from the app to obtain accurate proximity estimations. However, in order to mitigate the non-linearity between the RSS value and the distance of the emitting device, state estimation filtering techniques are applied, in order to provide better proximity estimation.

The most common and simplest filtering technique that we exploit is a simple moving average (SMA). This is an arithmetic moving average calculated by adding recent RSS values, and dividing that value by the number of time periods in the calculation average *N*. Even if this is the most adopted filtering technique, it is not enough to stabilize the RSS values. To this purpose, we introduce the *p*-percentile filter [32]. Generally, this approach is adopted in computer vision to remove outliers without degrading the resulting image. Differently, in this work, we use such a filter to minimize the variance of the RSS values so that to obtain stables values during the visiting path. More formally, given a set of beacons $B_{i}=\left\{b_{1},b_{2},\ldots ,b_{i}\right\}$ and a sequence of noisy RSS values $RSS_{i}\left(k\right)=\left\{rss_{i}\left(0\right),rss_{i}\left(1\right),\ldots ,rss_{i}\left(k\right)\right\}\;\forall i\in B_{i}$, the *p*-percentile filter is given as:

(1) $$RSS_{i}^{p}=P\left(RSS_{i}\left(k\right)\right)\;\forall i\in B_{i}$$

The *p*-th percentile is thus that value $RSS_{i}^{p}$ such that *p* percent of the $RSS_{i}\left(k\right)$ measures have a value lower than or equal to $RSS_{i}^{p}$.

The *p*-percentile filter is implemented to be used as a simple and energy-saving filtering technique, in comparison with other techniques. Through experimentation, it was found that a window size of 20 measures, i.e., k=20, equivalent to 10 s considering a 2 Hz beacon advertising frequency, is ideal in our application scenario.

We estimate the proximity between a visitor and a reference artwork over time for *k* observations, px(1),px(2),…,px(k), given a sequence of noisy RSS values $RSS_{i}\left(k\right)$, by using two energy-saving algorithms.

*Distance-based Proximity Technique*: The goal of the following algorithm is to infer the artworks nearest to the user by exploiting the RSS of the beacons installed close by the artworks. The idea of this algorithm is to convert the RSS values into distance for each beacon and, later, choose the beacon representing the closest artwork. We chose the path loss model [33] in order to model the relationship between RSS and distance:

(2) $$RSS=RSS_{0}-10nlog_{10}\left(d/d_{0}\right),\;d>d_{0}$$

where $d_{0}$ is the reference distance, such that the emitter and the receiver are always in line of sight (typically 1 m), $RSS_{0}$ is the RSS at a reference distance $d_{0}$, and *n* is the path loss exponent that regulates how severe is the attenuation in a given environment. The distance function between each artworks *i* and the user can be estimated as:

(3) $$f_{i}\left(d\right)=e^{-\frac{RSS_{i}^{p}-RSS_{0}}{10n}}\;\forall i\in B_{i}$$

The nearest artwork px(k) at time *k* is given by

(4) $$px=\underset{d}{\mathrm{argmin}}\;f_{i}\left(d\right)$$

while a ranking of the “most near” artworks could be easily generated by sorting Equation (3). It is worth noticing that finding the minimum distance of a decreasing function such as Equation (2) is equivalent of choosing:

(5) $$px=\underset{i}{\mathrm{argmax}}\;RSS_{i}^{p}$$

*Threshold-based Proximity Technique*: Another simple and energy-saving algorithm we used in this work is based on fixing a range in meters which represents a reasonable value to visit artworks. The nearest artworks are given by:

(6) $$px_{i}=\;\forall i\;\exists \;RSS_{i}^{p}\geq RSS_{Th}$$

where $RSS_{Th}$ is the RSS value for which the user can be considered in the range. Unlike the distance-based proximity technique, this algorithm generates a list of possible nearest artworks.

## 4. Identifying Artwork Proximity with the Bluetooth Tags

We conduct a set of experiments to test the functionalities of the RE.S.I.STO app as well as to test the performance of the implemented proximity detection algorithms. We detail in this section the experimental settings (Section 4.1) and the obtained results (Section 4.2).

### 4.1. Preliminary Settings

We initially set up a preliminary data collection campaign whose goal is to verify that the mobile app correctly collects beacons without any abnormal interruption. In this case, our goal is to identify possible software/hardware issues reducing the amount of collected beacons. Such a preliminary assessment is obtained in a controlled environment without considering environmental conditions, such as obstacles, interferences, and any source of signal attenuation. We consider the following 3 test typologies:

- Stress test (**$T_{s}$**): the objective is measuring the maximum amount of collected beacons in a relatively short time period;
- Stability test (**$T_{st}$**): the objective is testing the consistency of the number of collected beacons for a long time period;
- Calibration test (**$T_{c}$**): the objective is calibrating the proximity detection algorithms to compute a reference benchmark for the RSS features.

**Figure 5:**
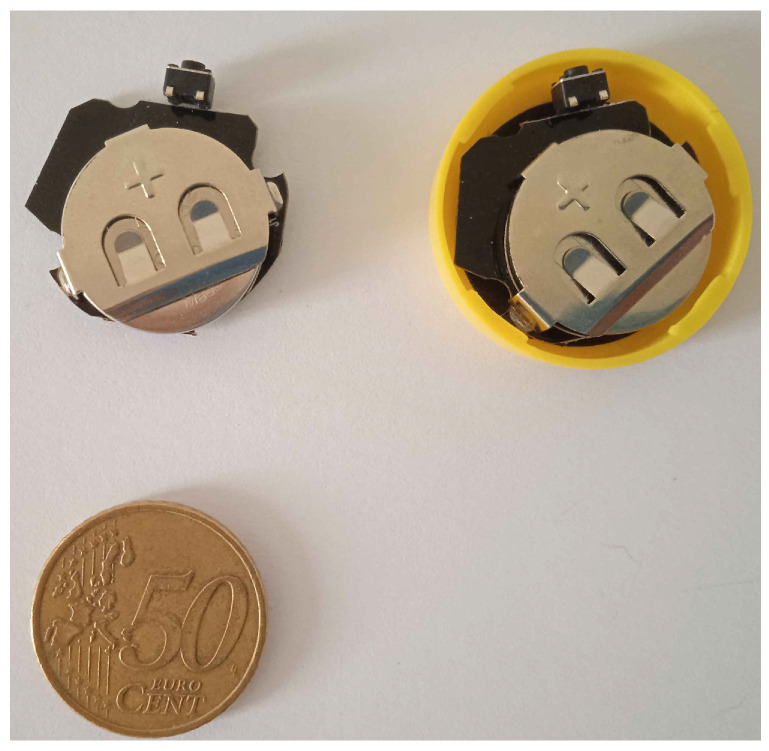
Bluetooth tags used during our experiments.

Tests are conducted with Bluetooth tags produced by GlobalTag and configured with power of transmission varying according to the test typology (e.g., from −23 dBm to 0 dBm). We set the tag advertising interval to 2 Hz. Tags are powered with a CC2032-type battery, with a battery level higher than 2.9 V. Tags are configured with the iBeacon payload, piggybacking the major number associated with the tag. Figure 5 shows the tags we used during our experiments.

During our data collection campaign, we use 2 smartphones to collect and store beacons:

- Honor 9 (**H9**) running Android 8, equipped with Bluetooth 4.2 chip-set;
- Google Pixel 4a (**GP**) running Android 12, equipped with Bluetooth 5.0 chip-set.

We report in Table 3 a summary of all the test settings.

#### 4.1.1. Stress Test

**Figure 6:**
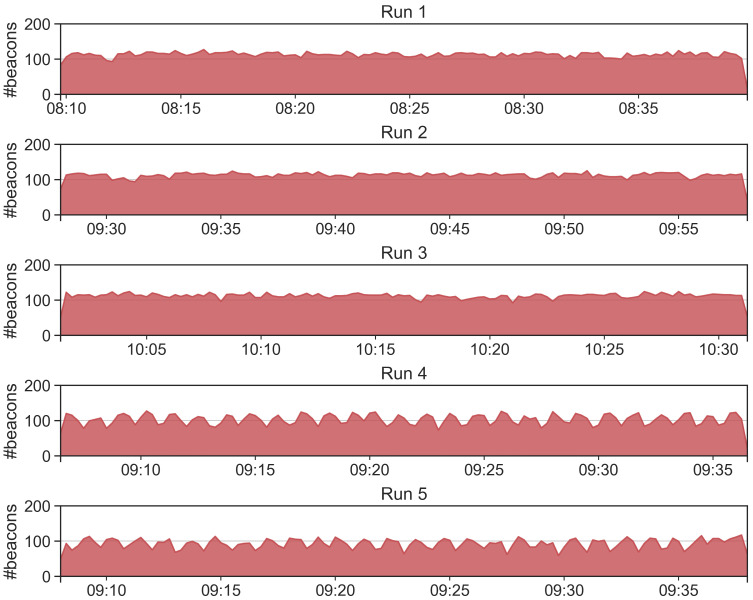
Number of beacons collected in $T_{1}$ for each run.

The purpose of the $T_{1}$ test is measuring the maximum number of beacons that can be collected and, in turn, to derive the beacon loss rate. Such rate is a crucial aspect for the proximity detection, as it determines the expected number of beacons to analyze. We repeat $T_{1}$ for 5 runs, each run lasts for 30 min. The tags and the receiving devices are positioned on a desk at a distance of approximately 50–70 cm. Results are reported in Table 4. Results of $T_{1}$ show that the average beacon loss rate is about 28% for the 5 tags used, a value far below 50% that guarantees the possibility of successfully running the proximity detection algorithms. We refer to [34,35] for a real-world Bluetooth beacon dataset in which we describe some issues in collecting data with an Android native mobile application. We also analyze if the beacons are collected continuously in time or if the RE.S.I.STO app receives data in burst. We report in Figure 6 a time series for each of the 5 runs showing the amount of collected beacons in a time window of 15 s. From the figure, it is possible to observe the continuous monitoring of the app without interruptions for each of the runs.

#### 4.1.2. Stability Test

**Figure 7:**
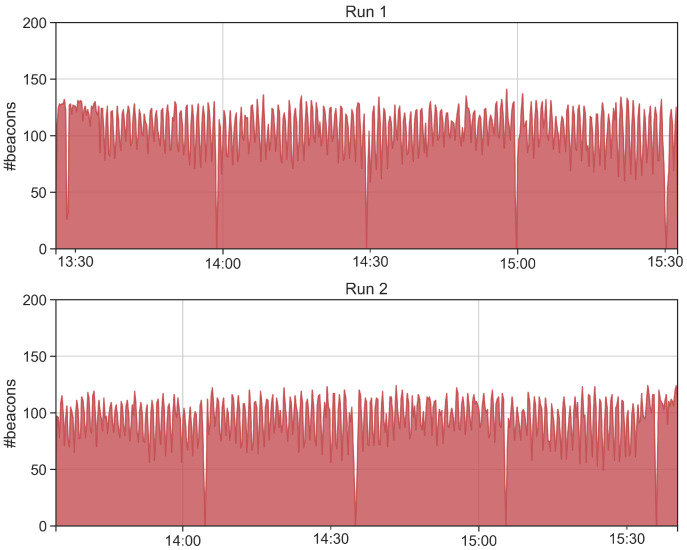
Cumulative number of beacons collected in $T_{2}$ for each run.

**Figure 8:**
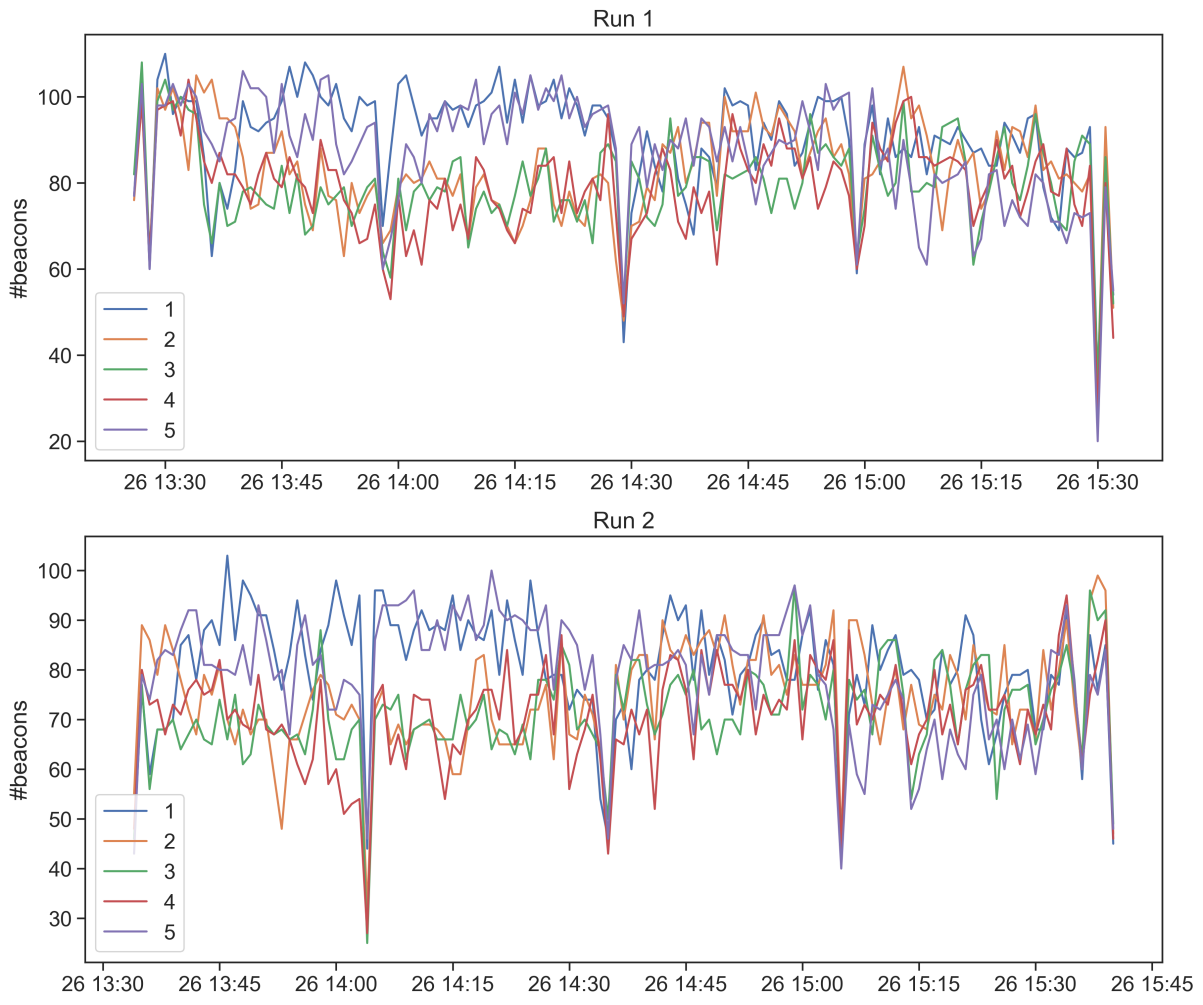
Number of beacons collected in $T_{2}$ for each run and for each of the 5 tags.

Concerning stability tests $T_{2}$ and $T_{3}$, the purpose is collecting beacons for a long time period, so that to assess the stability of the data collection. In particular, test $T_{2}$ is composed by 2 runs executed with H9 and GP devices, for a total monitoring time of 2 h. Differently, test $T_{3}$ is composed by 1 single run executed with GP device for a total monitoring time of 5 h. The tags and the receiving devices are positioned on a desk at a distance of approximately 50–70 centimeters. We first report the number of collected beacon and the beacon loss rate and, then, we analyze the obtained time series. Table 5 reports the obtained results for both of the tests. In these cases, the beacon loss rate is below 50%, $T_{2}$ reports an average beacon loss rate of 33.3%, while $T_{3}$ of 16%. We then analyze the time series of the collected beacons in order to identify anomalies during the beacon collection. Figure 7 and Figure 8 show respectively the number of collected beacons aggregated in a time window of 15 s and the number of collected beacons for each of the 5 tags aggregated in a time window of 60 s. As a general trend, the app is able to continuously collect beacons without any significant interruption. However, we notice four time intervals during which the number of collected beacons drops. Such interruptions are caused by the stopping of the Kontaktio React Native library used to gather beacons. We observe that after 30 min of monitoring, the library does not collect any more data. We fix this issue by programmatically restarting the Beacon Logger service after its interruption. We report in Figure 8 the number of collected beacons for each of the five tags. In addition, in this figure, it is possible to observe the previously described issue. In particular, as soon as the app stops collecting data, the number of received beacons drops for all the tags, confirming that the problem is not caused by tag malfunction.

**Figure 9:**
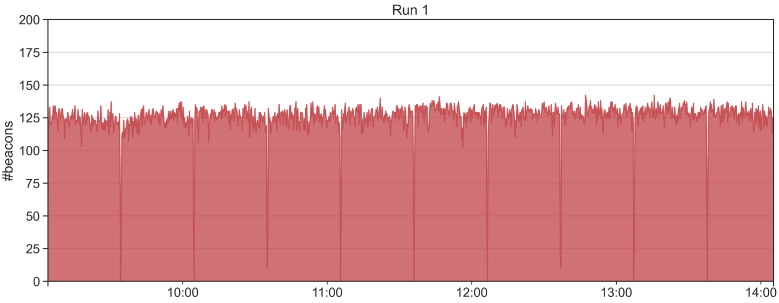
Cumulative number of beacons collected in $T_{3}$.

Similar considerations also apply for the $T_{3}$ test. Differently from $T_{2}$, in this case, we execute a long-lasting run of 5 h, as shown in Figure 9. We still observe several interruptions during the beacon collection that slightly reduce the expected number of beacons. However, such interruptions are restricted to the order of a few seconds.

#### 4.1.3. Calibration Test

The purpose of the calibration test $T_{8}$ is analyzing the variation of the beacon’s RSS at stationary conditions. More specifically, we position 5 tags, configured with a transmission power of −23 dBm and 2 Hz advertisement frequency, in vertical position at 1.5 m distance from the receiving device. We use the GP device to collect the beacons for a total test duration of 10 min.

We first compute the number of collected beacons and we derive the beacon loss rate. Then, we analyze the aggregated RSS distribution and the same distribution split for each of the tags. Table 6 reports the number of collected beacons and the beacon loss rate. We observe that the obtained values match with the test results in $T_{1}\cdots T_{4}$ (reported in Table 4 and Table 5). In particular, the average number of collected beacons in $T_{4}$ is 1048 with a beacon loss rate of 15%.

**Figure 10:**
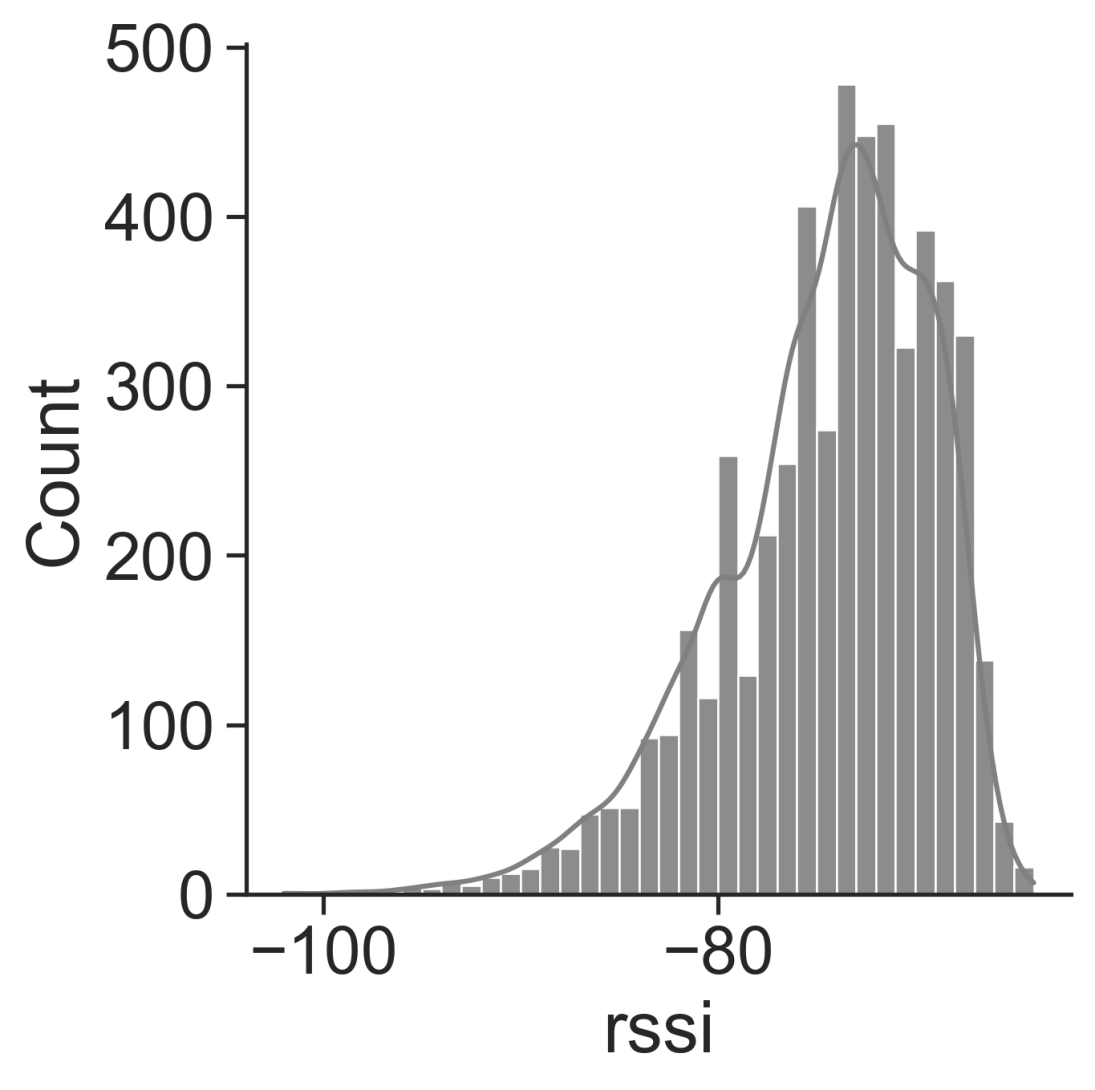
Cumulative distribution of beacon’s RSS for $T_{8}$.

We show in Figure 10 the distribution of the beacon’s RSS. The distribution peaks at −74.6 dBm (such value is used for the configuration of the Threshold algorithm), with a standard deviation of 5.3 dBm, 25-th percentile of −78 dBm and 75-th percentile of −71 dBm.

**Figure 11:**
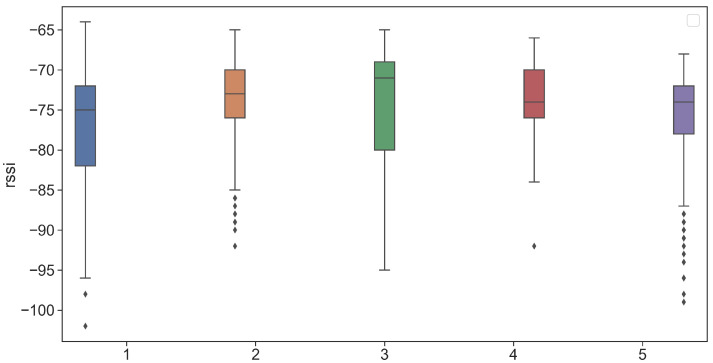
Box plots of the beacon’s RSS distribution for $T_{8}$.

**Figure 12:**
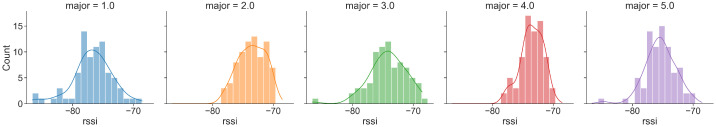
Distributions of beacon’s RSS for $T_{8}$ for each tag.

Although the RSS distribution provides a reference value for determining the proximity between the visitor and the artwork, we observe a notable dispersion of the RSS values. To this purpose, we further investigate the RSS values estimated for single tags. We report in Figure 11 and in Figure 12 the RSS distributions for the 5 tags. From the figures, we observe that the range of the RSS values varies from −102 dBm (tag with major number 5) to −64 dBm (tag with major number 1).

As representative examples, tags with major numbers 1 and 4 remarkably differ. In the first case, the RSS distribution shows a mean and standard deviation of −76.7 dBm and 6.4 dBm, respectively, while in the second case of −73.5 dBm 3.89 dBm, respectively.

**Figure 13:**
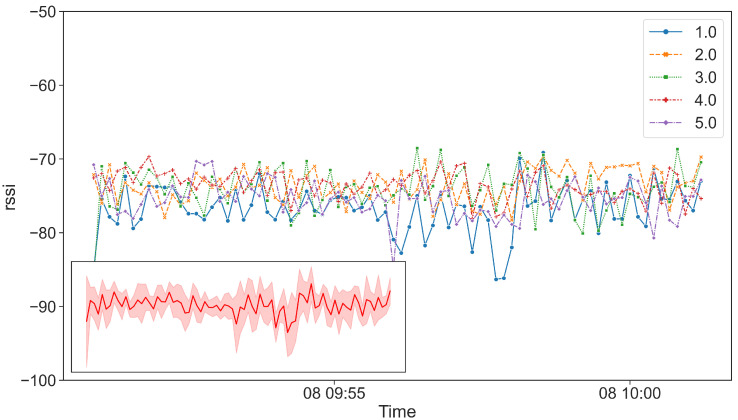
RSS time series of 5 tags.

We finally analyze the time series of the tag’s RSS in order to better understand the variations reported in Figure 11 and Figure 12. Figure 13 shows the RSS time series of the 5 tags. Time series are 8-s re-samples so that to recognize the different tags and to observe the RSS fluctuations. From the figure, we recognize an oscillating pattern with increasing/decreasing values, however the RSS range varies for each tag in a different dBm interval. Furthermore, we report in Figure 13 an inset showing the aggregated time series with a red band reporting the aggregated RSS standard deviation.

The previous analysis clearly shows a strong variation of RSS value for different tags. According to the Bluetooth Low Energy specification (https://www.bluetooth.com/specifications/specs/core-specification/ (accessed on 1 September 2021)), tags can advertise beacons on different channels. More specifically, the channels used for beaconing range in the following frequencies: 2.402 Ghz (channel 37), 2.426 Ghz (channel 38) and 2.480 Ghz (channel 39). Generally, emitting devices select the channel following a Round-Robin scheduling (referred to as frequency hopping), starting from channel 37. The channel used for propagating the beacon messages affects the received signal strength on the receiving device, giving rise to the pattern reported in Figure 13. To the best of our knowledge, the Android API does not provide any channel information, therefore, it is not possible to filter out beacons emitted only on a specific channel, as discussed in [36].

### 4.2. Experimental Results

We now detail how we conduct our experimental tests in order to assess the performance of the implemented proximity detection algorithms in two pilot sites reproducing a typical museum experience. A museum is generally organized with a suggested path designed to guide a visitor through the exposed artworks. However, the proposed path is not mandatory; hence, visitors are also allowed to alter the order of the visited artworks. Moreover, since the goal of the museum supervisor is to highlight the beauty of the exposed artworks, the artworks are typically well-spaced so that visitors can enjoy the museum experience.

To this purpose, we identify two pilot sites in which we deploy Bluetooth tags for detecting proximity with artworks. More specifically, we test our solution in:

- Pilot 1: a wide open space of approximately 190 m${}^{2}$ located in our research institute;
- Pilot 2: Camposanto Monumentale of Pisa, located in Piazza dei Miracoli, Pisa (Italy).

For what concerns Pilot 1, the selected open space well reproduces an indoor artwork collection deployed in a single room, in which visitors are free to move and get in proximity of the artworks. In this pilot site, visitors can only observe artworks with face-to-face orientation, meaning that it is not possible to watch the scene from behind of sideways. Differently, Pilot 2 is characterized by an open gallery bounding a central cloister. The exposed artworks are all positioned below the open gallery. Visitors are free to move and watch the scene from different perspectives. As an example, the exposed half-length statues are installed on a stand and visitors can turn around the stand. This aspect, further increases the complexity of the proximity detection because we cannot assume to deploy the Bluetooth tags with a specific orientation with respect to the visitor. Pilot 2 comprises different typologies of artworks, for the purpose of this work, we considered: half-length statues, altars, sarcophagus and wall slabs.

**Figure 14:**
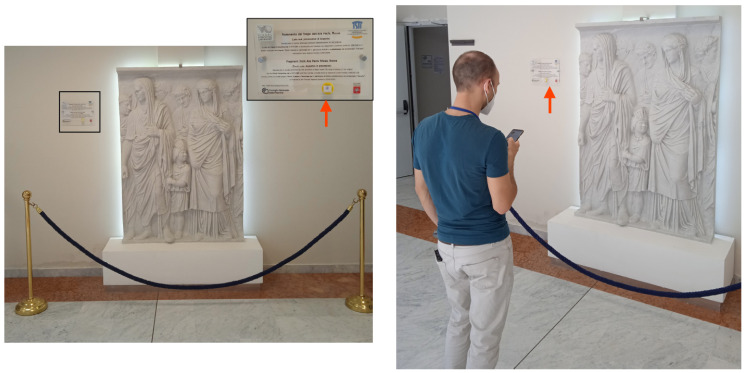
Artwork visit and deployment of a BLE tag in Pilot 1.

**Figure 15:**
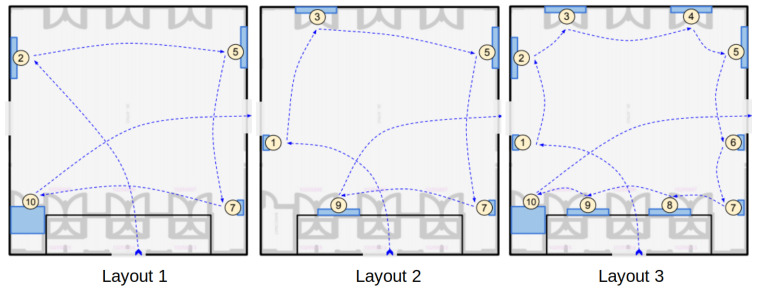
The three visiting layouts for Pilot 1.

**Figure 16:**
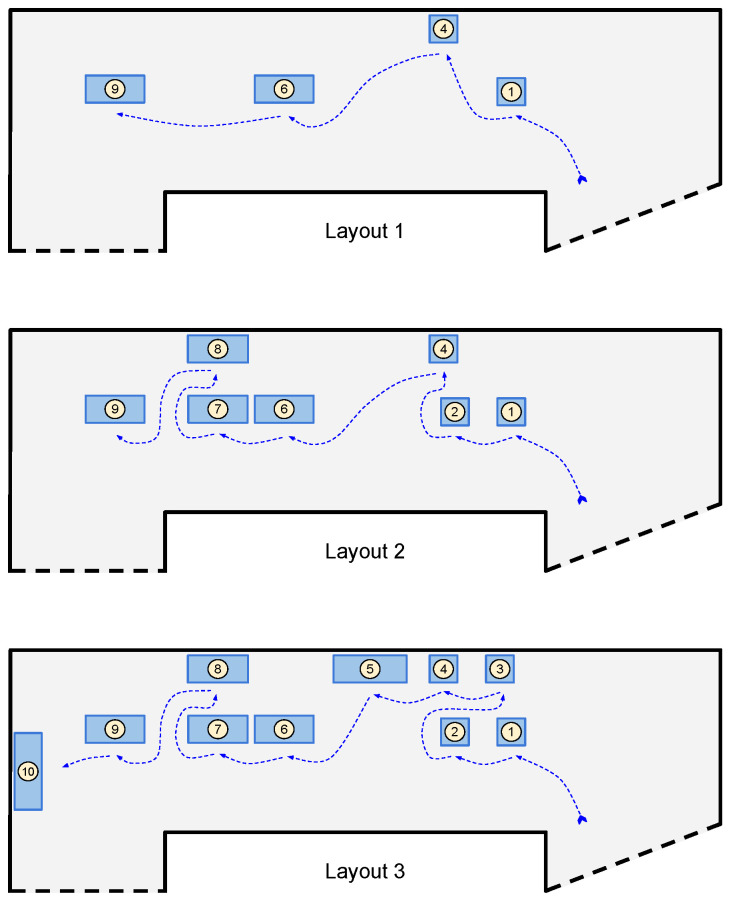
The three visiting layouts for Pilot 2.

All the artworks are associated with a Bluetooth tag (with major numbers ranging from 1 to 10), and we assume that each visit consists of 2 min at 1.5 m distant from the artwork, as shown in Figure 14. The visitor moves from an artwork to the next one with a pedestrian speed of approximately 1.8 m/s. Tags are located at 1.5 m from the ground and they are set with a power of emission of −23 dBm at 2 Hz and a battery level of at least 80%. We use the GP (Google Pixel 4) as receiving device and we test different visiting layouts for the two pilot sites. In particular, as reported in Figure 15 and Figure 16, we considered:

- Layout 1: 4 artworks for pilot 1 and 2;
- Layout 2: 5 artworks for pilot 1 and 7 artworks for pilot 2;
- Layout 3: 10 artworks for pilot 1 and 2.

During the visits, the visitor holds the GP device in hand and we collect both the output of the proximity algorithms (Max and Threshold) and the ground truth (GT), namely the starting and ending time of an artwork’s visit. It is worth noticing that we calibrate the Threshold algorithm only once. More specifically, from the calibration analysis reported in Section 4.1.3, we conclude that setting a threshold to −75 dBm provides the optimal performance. We adopt such setting in pilot 1 and 2 in order to verify the robustness of such setting at very different conditions.

The ground truth is used to evaluate the outcome of the implemented proximity algorithms. As described in Section 3.1, the app performs two main tasks: collecting beacons for a time window of Δ seconds and analyzing the collected data so that to detect the artwork proximity. As a result, the GT and the output might result with de-synchronized timestamps. In order to temporally align the GT with the output of the algorithms, we re-sampled the two time series so that to have at each timestamp (one for each second) both the GT and the algorithm’s results. We compute two well-adopted metrics to evaluate a classification process, namely: Accuracy (Ac) and F1 score (F1, obtained as a combination of the Precision and the Recall metrics) [37].

As some errors during the proximity detection might be possible, we implement our proximity algorithms to return not only the most probable artwork in proximity, but also the second and the third artwork in proximity. More specifically, the result is a vector of three items: $\left[a_{1},a_{2},a_{3}\right]$ corresponding to the first, second and third artwork. In this way, we are able to compute the performance by considering three options. Finally, in order to mitigate the negative effect of the frequency hopping discussed in Section 4.1.3, the proximity algorithms consider a specific statistic of the beacons collected from each tag. More specifically, our objective is being able to consider only beacons emitted on a specific channel, as they are supposed to have lower RSS variations (see time series in Figure 13). Given *B*, the beacons received from tag *j* during the time window [t,t+Δ] (see Section 3.1), we compute the 75th percentile of the beacon’s RSS of set *B*. Such statistic allows us to consider only the left-side of the RSS distributions reported in Figure 12, hence, we only consider beacons with higher RSS values.

**Figure 17:**
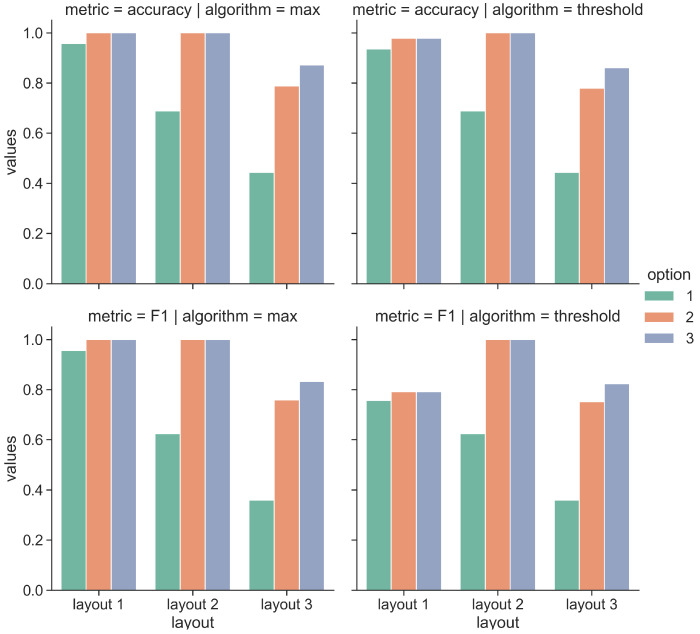
Performance results of the proximity algorithms with pilot 1.

**Figure 18:**
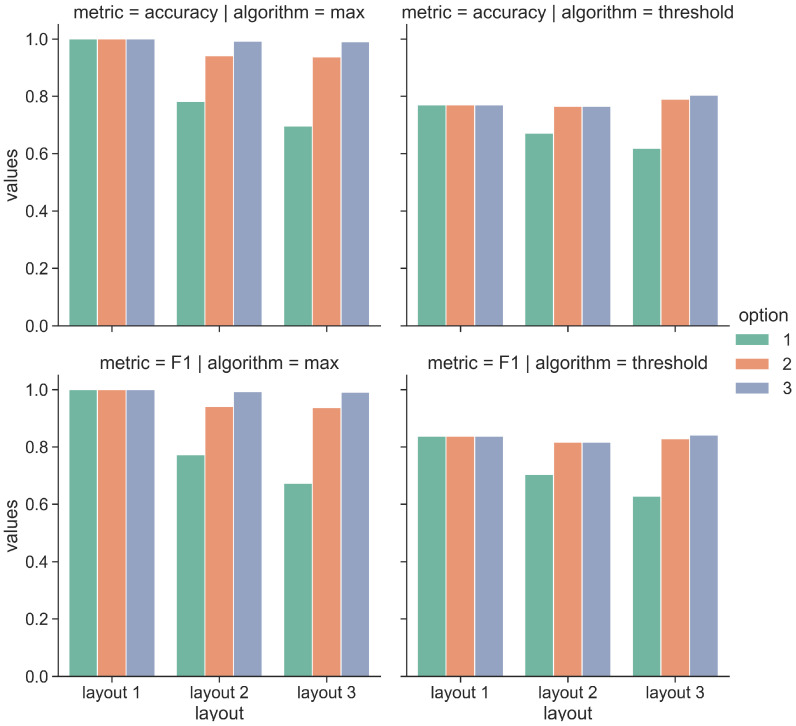
Performance results of the proximity algorithms with pilot 2.

Figure 17 and Figure 18 show the obtained results for pilot 1 and 2, respectively. We report the evaluation metrics in the rows, while in the columns, we report the implemented algorithms. The bar plots inside each graph show the metric values by varying two settings:

- The option: we compute the metrics by considering if the correct artwork is contained in the first option, in the first 2 options or if it is contained in the 3 options;
- The layout: we compute the metrics by considering 3 layouts of increasing complexity.

Concerning the pilot 1 reported in Figure 17, we observe that by increasing the number of considered options, the obtained results increase for all the layouts and for the two implemented algorithms. More specifically, the max algorithm provides the best results for all the layouts. When we consider only the first option with layout 1, we obtain an accuracy and F1 score of 0.95, while with the second and the third option, with layout 1, we obtain perfect accuracy and F1 scores. Differently, the performance degrades with more complex layouts, such as layouts 2 and 3. With layout 2 and with the first option, the accuracy decreases to 0.68 and F1 to 0.62, while when we consider options 2 and 3, the accuracy and F1 score still are perfect. Finally, layout 3 is more challenging. As reported in Figure 14, we consider 10 artworks during the museum visit. Some of the artworks are in close proximity, such as the pairs (#1, #10) and (#10, #9). When we consider only the first option, the accuracy value decreases to 0.4 and F1 to 0.3, while when considering the options 2 and 3, the obtained accuracies and F1 values are 0.78 and 0.7 and 0.98 and 0.83, respectively. We observe a similar trend for the values obtained with the Threshold algorithm. We now analyze the results obtained for pilot 2 reported in Figure 18. In this case, the performance tends to decrease by increasing the complexity of the tested layout. However, we observe that the obtained results (in terms of Accuracy and F1 score) are generally higher with respect to pilot 1. In particular, the Accuracy score returned by the Max algorithm with layout 3 is 0.69 considering option 1, 0.93 with option 2 and 0.99 with option 3. The Max algorithm also provides strong results with layouts 1 and 2, as expected in these cases, the accuracy scores are higher than the ones obtained for the most challenging layout. Similar considerations also apply for the Threshold algorithm. In this case, the Accuracy score with layout 3 is 0.61 considering option 1, 0.79 with option 2 and 0.8 with option 3. We also observe that the Threshold algorithm does not reach perfect Accuracy in none of the layouts and the options.

## 5. Discussion and Conclusions

Location-based services offer the possibility of delivering functionalities to end-users by inferring their location or the proximity with specific points of interest. In this paper, we present the design and a quantitative assessment of the reliability and performance of the RE.S.I.STO application, which is used to automatically detect the proximity between visitors and artworks. In particular, we present the RE.S.I.STO app, which is targeted to visitors of museums in the medieval city of Pisa (Italy). We first present the design of our cross-platform mobile application, based on the React-Native framework. We detail the architectural design and how we logically split the implemented software modules to collect sensing information and to elaborate on it for the purpose of detecting the artwork proximity. Our mobile application relies on the Bluetooth Low Energy protocol and, specifically, on the iBeacon technology. We implement two proximity algorithms and we detail our experiments composed of stress, stability and calibration tests. We show the performance of the app both in terms of beacon loss rate, accuracy and F1 scores. In particular, our stress and stability tests demonstrate the robustness of the app during the beacon collection task. We test the app with long-lasting session (up to 5 h of continuous monitoring) and we measure the ratio between expected and collected beacons. We then analyze the performance of the app during the proximity detection. This work also investigates some issues related to the calibration caused by the use of multiple advertising channels which imply a high variation of the RSS values at fixed distance. We also discuss how to mitigate the RSS fluctuations by computing a statistic of the received beacons’ RSS. To this purpose, we reproduce three typical museum layouts in a wide indoor environment and we compare the artwork ground truth with the outcome of the algorithms. Our results show that we can successfully detect the correct artwork with an accuracy up 95% for layout 1 and by considering the first option provided by the app. As well reported in Table 2, the proposed solution has been validated in a realistic scenario with a nontrivial experimental path. These features are also taken into consideration by [7,8], while [18,30] evaluated the system performance in a more controlled environment such as a laboratory. Moreover, as in [8], we validated the performance results by using different mobile devices, but usually this feature is not analyzed in the literature. The use of commercial devices and the robustness test we conducted are other characteristics of the proposed system that are usually not evaluated and reported in the literature [9,10]. The table has been defined by considering those features, making the proximity detection a challenging task in indoor environments, such as the complexity of the path, the realistic scenario and the adopted devices to estimate the signal strength of beacon messages. We then designed our system trying to address such features as compared in Table 2. Our experimental settings demonstrate all the complexity behind the use of Bluetooth tags for the proximity detection. In particular, we observe a high variability of the received signal strength of beacons emitted by commercial tags at similar condition. Such variability is caused not only by environmental conditions (obstacles, fading and signal reflections), but also from the use of multiple advertising frequencies. As a result, we experience values of RSS ranging in a considerable dBm interval. We discuss in the paper how we mitigate such a behavior. However, we argue that more advanced techniques can be used to further increase the performance of our algorithms. We consider two possible lines of investigation. On the one hand, the use of data fusion techniques enables us to merge information obtained from heterogeneous sensors such as in inertial and proximity sensors. Such data can be elaborated with filtering techniques so that to filter out noisy data and improve the identification of the closest artwork. Furthermore, knowledge of the indoor map can be exploited to contain the errors by avoiding unfeasible outcomes of the mobile app. Moreover, data fusion techniques can be also applied to image sources. More specifically, we consider the possibility of using 3D indoor maps as a reference background and to augment them with contextual information about the artwork a visitor is looking at. These experiences can be further enriched by using 3D visors, e.g., Epson Moverio as an alternative rendering device. On the other hand, promising mobile technologies can increase the performance of the proposed solutions. We argue that the Bluetooth 5.1 protocol, introducing two key-features, namely the angle of arrival (AoA) and the angle of departure (AoD), will allow to obtain a significance reduction of the localization error, reshaping the context of location-based services and the visitor’s experience. Another technology that we believe is useful to point out and which could revolutionize the next generation proximity systems is UWB. Leveraging the time-of-flight (ToF) or the time-difference-of-arrival (TDoA) measured between the mobile and the UWB tags, we argue that this technology could increase the accuracy in all the indoor localization fields. 

## Figures and Tables

**Table 1:** Summary of the main proximity detection technologies used in the literature.

Technology	Main Advantages	Main Limitations	Related Works
BLE	Widely diffused, low power consumption	Requires the physical deployment of the tags, RSS is affected by both crowd and signal reflections	[7,8,9,10]
WiFi	Widely diffused, no dedicated infrastructure required	Low proximity detection accuracy	[6,11,12,13,14,15]
UWB	Reaches few centimeters of accuracy	Requires an extensive infrastructure setup, compatible end-user devices are still not very diffused	[16,17,18]
NFC	Highly available on market devices	Restricted interaction in crowd environments	[19,20,21]
Visual detection	Intuitive user experience	Extensive training phase required, susceptible to partial visual occlusion	[6,22,23]
Bar/QR Codes	Cheap technology, easy to deploy	Might interfere with the artwork visual, taking a photo could be prohibited in the museums	[24,25]
Ultrasonic	No dedicated hardware on the user’s mobile device, signals can be generated with off-the-shelf speakers	Requires the infrastructure deployment, low accuracy	[25,26]
Infrared	Cheap technology	Highly directional beams, requires line-of-sight, many devices lacking of IR transceivers	[27,28,29]

**Table 2:** Comparison of selected BLE proximity detection solutions for indoor museums.

	Realistic Scenario	Device Heterogeneity	Complex Path	Commercial Devices	Robustness Tests	Preliminary RSS Data Analytics	Real-Time Outcome
Martella, C. et al. [7]	YES Cobra Museum of Modern Art (NL)	NO Single device model used	YES Several artworks spread in multiple museum rooms	NO Ad-hoc solutions for both the anchors and tags	NO Device performance are known	YES Filtering pipeline to handle bursty and noisy data	NO
Yoshimura Y. et al. [8]	YES Louvre Museum (FR)	YES Several visitors’ devices detected	YES Points of interest along the museum rooms	NO Custom device employed	NO Not considered	NO Not considered	NO
Kikuchi K. et al. [9]	NO Lab environment	NO Only laptop PC used	NO Few positions tested	NO Experimental BLE transmitter	NO Very short testing period	YES Triangulation by exploiting beacon’s Direction-of-Departure	YES
Allurwar N. et al. [10]	NO Lab environment	NO Single device model used	NO Small lab test	NO Ad-hoc solutions for the BLE tags	NO Not considered	NO Not considered	YES
Jiménez A.R. et al. [18]	NO Lab environment	NO Single device model used	NO Few points of interest in a corridor	YES Estimote beacons	NO Not considered	YES Path-loss model fitting and bias correction	YES
Spachos P. et al. [30]	NO Lab environment	NO Single device model used	NO Few positions tested	YES Gimbal Series 21 beacons	NO Not considered	YES Path-loss model fitting and Kalman filtering	YES
Proposed solution	YES Camposanto Monumentale of Pisa (IT)	YES Multiple devices used	YES Artworks along a path in the museum	YES GlobalTag beacons	YES Duration and stress test performed	YES Beacon RSS distribution analysis for channel hopping mitigation	YES

**Table 3:** Overview of the experimental data collection campaign.

Test ID	Type	Runs	Devices	Duration (min)	Adv. Freq. (Hz)	Power (dBm)	Tags
$T_{1}$	$T_{s}$	5	2(H9,GP)	150	2	0	5
$T_{2}$	$T_{st}$	1	2(H9,GP)	252	2	0	5
$T_{3}$	$T_{st}$	1	1(GP)	301	2	0	5
$T_{8}$	$T_{c}$	1	1(GP)	10	2	−23	5

**Table 4:** Results of stress test $T_{1}$.

Beacon ID	#Beacons	Beacon Loss Rate
1	13,427	25%
2	12,123	32%
3	12,090	32%
4	13,251	26%
5	13,314	26%
**avg**	**12,841**	**28%**

**Table 5:** Results of stability tests $T_{2}$ and $T_{3}$.

	Test $T_{2}$		Test $T_{3}$	
**Beacon ID**	**#Beacons**	**Beacon Loss Rate**	**#Beacons**	**Beacon Loss Rate**
1	21,737	28.2%	30,378	15.9%
2	19,953	34.1%	30,071	16.8%
3	19,157	36.7%	30,029	16.9%
4	19,027	37.1%	30,112	16.7%
5	21,053	30%	30,447	15.8%
**Avg**	**20,185**	**33.3%**	**30,207**	**16.4%**

**Table 6:** Results of calibration test $T_{8}$.

Beacon ID	#Beacons	Beacon Loss Rate
1	1025	17.2%
2	1055	14.8%
3	1050	15.2%
4	10,741	13.3%
5	10,384	16.2%
**Avg**	**1048**	**15%**
